# The metabolic cost of lowering blood pressure with hydrochlorothiazide

**DOI:** 10.1186/1758-5996-5-35

**Published:** 2013-07-09

**Authors:** Angela L Price, Ildiko Lingvay, Edward W Szczepaniak, Jaime Wiebel, Ronald G Victor, Lidia S Szczepaniak

**Affiliations:** 1 Department of Internal Medicine, UT Southwestern Medical Center, Dallas, Texas, USA; 2 Cedars -Sinai Medical Center, The Heart Institute, Los Angeles, California, USA

**Keywords:** Type 2 diabetes, Valsartan, Hydrochlorothiazide, Proton magnetic resonance spectroscopy, Insulin sensitivity, Insulin secretion

## Abstract

**Background:**

The landmark Antihypertensive and Lipid-Lowering treatment to prevent Heart Attack Trial (ALLHAT) placed a new spotlight on thiazide diuretics as the first-line therapy for hypertension. This is concerning as thiazide-diuretics may contribute to comorbidities associated with the current epidemic of obesity. Previous randomized clinical trials have linked thiazide diuretic treatment to insulin resistance, metabolic syndrome, and increased incidence of type 2 diabetes.

**Methods:**

This proof of concept, longitudinal, randomized, double–blind study evaluated the effects of the angiotensin II receptor blocker Valsartan and the specific thiazide diuretic Hydrochlorothiazide (HCTZ) on hepatic triglyceride level (primary outcome), as well as triglyceride levels within other organs including the heart, skeletal muscle, and pancreas. Additionally, we evaluated whether myocardial function, insulin sensitivity, and insulin secretion were affected by these treatments.

**Results:**

Hepatic TG levels increased by 57% post HCTZ treatment: ∆hTG _HCTZ_ = 4.12% and remained unchanged post Valsartan treatment: ∆hTG _V_ = 0.06%. The elevation of hepatic TG levels after HCTZ treatment was additionally accompanied by a reduction in insulin sensitivity: ∆SI _HCTZ_ = -1.14. Treatment with Valsartan resulted in improved insulin sensitivity: ∆SI _V_ = 1.24. Treatment-induced changes in hepatic TG levels and insulin sensitivity were statistically significant between groups (p_hTG_ = 0.0098 and p_SI_ = 0.0345 respectively). Disposition index, DI, remained unchanged after HCTZ treatment: ∆DI _HCTZ_ = -141 but it was increased by a factor of 2 after treatment with Valsartan: ∆DI _V_ =1018). However, the change between groups was not statistically significant. Both therapies did not modify abdominal visceral and subcutaneous fat mass as well as myocardial structure and function. Additionally, myocardial, pancreatic, and skeletal muscle triglyceride deposits remained unchanged in both therapeutic arms.

**Conclusions:**

Our findings are two-fold and relate to hepatic steatosis and insulin sensitivity. HCTZ treatment worsened hepatic steatosis measured as hepatic triglyceride content and reduced insulin sensitivity. Valsartan treatment did not affect hepatic triglyceride levels and improved insulin sensitivity. The results of this study reinforce the message that in patients at risk for type 2 diabetes it is particularly important to choose an antihypertensive regimen that lowers blood pressure without exacerbating patient’s metabolic profile.

## 

The incidence of obesity and obesity-related complications such as hypertension and type 2 diabetes are rising steadily despite the increased public and scientific awareness of this multifactorial problem. Although specific efforts to turn the obesity tide concentrate on the development of new treatment strategies, it is important to revisit old therapies and review their side effect profiles as some treatments may silently augment the metabolic syndrome.

The landmark Antihypertensive and Lipid-Lowering treatment to prevent Heart Attack Trial (ALLHAT) placed a new spotlight on thiazide diuretics as the first-line therapy for hypertension [1].This is concerning as thiazide-diuretics may contribute to comorbidities associated with the current epidemic of obesity. Previous randomized clinical trials have linked treatment with thiazide diuretic to insulin resistance, metabolic syndrome, and increased incidence of type 2 diabetes [2,3].

On the contrary, evidence accumulates that therapies which interfere with the adverse metabolic effects of angiotensin II, such as angiotensin II receptor blocking (ARB) or/and angiotensin converting enzyme (ACE I) therapies, cause no metabolic harm as confirmed by the DREAM [4] and NAVIGATOR [5-7] studies. The favorable metabolic action of ARB and ACE-I agents could originate from improvement of insulin sensitivity [8] or could be facilitated through the recruitment and differentiation of adipocytes [9]. Both mechanisms could lead to reduction in ectopic deposition of triglyceride in organs such as liver, heart, pancreas and skeletal muscle, a hypothesis that has not yet been tested.

We present the results of a randomized study comparing the metabolic effects of treatment with hydrochlorothiazide (HCTZ) and Valsartan in individuals at high risk for development of type 2 diabetes. We specifically evaluated the effect of these treatments on intra-hepatic triglyceride content as well as insulin sensitivity, beta-cell function, and ectopic triglyceride deposition in the heart, pancreas, and skeletal muscle.

## Methods

This proof of concept, longitudinal, randomized, double–blind study evaluated two antihypertensive treatments in individuals at high risk for diabetes. The study was registered as clinical trial # NCT00745953. The research protocol was approved by Institutional Review Board at UT Southwestern Medical Center. All participants gave informed written consent prior to experiments.

Our objective was to compare the effects of the angiotensin II receptor blocker Valsartan and the thiazide diuretic Hydrochlorothiazide (HCTZ) on hepatic triglyceride level (primary outcome), as well as triglyceride levels within other organs including the heart, skeletal muscle, and pancreas. Additionally, we evaluated whether myocardial function, insulin sensitivity, and insulin secretion were affected by these treatments.

### Study subjects

Eighty-two individuals were screened for eligibility to participate in the study. Qualifying individuals were young adults (age range 18–55 years)with 3 of the following 5 conditions: fasting glucose > 100 mg/dl; waist circumference: men > 102 cm, women >88 cm; HDL: men < 40 mg/dl, women <50 mg/dl; TG > 150 mg/dl; BP > 130/85 mm Hg. Individuals with a previous diagnosis of type 2 diabetes, stage 2 hypertension (BP > 160/110 mm Hg), or those exposed to thiazolidinediones, statins, diuretics, ARB, ACEI, or any investigational agents within 6 months prior to the study did not qualify. Claustrophobia and presence of metallic implants in the body were magnetic resonance imaging (MRI) exclusion criteria. Additionally, women of child bearing age who were not using reliable contraception or those breast feeding did not qualify.

**Figure 1:**
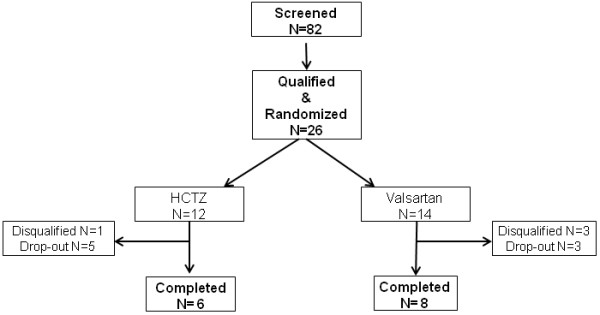
Study consort diagram.

Twenty-six individuals who qualified and agreed to participate were randomized in blocks of 4 to either therapy (12 to HCTZ and 14 to Valsartan). Eight individuals (5 in HCTZ and 3 in Valsartan) did not complete the protocol for various personal reasons. Results from 4 individuals who completed therapy (1 in HCTZ and 3 in Valsartan) were excluded from analysis due to either significant lifestyle changes (N = 1) that resulted in a large amount of weight loss or because individuals were not available for the end-of-study evaluation (N = 3). Fourteen individuals (6 in HCTZ and 8 in Valsartan) completed all study procedures and were considered in final analysis (Figure 1).

### Experimental protocol

Qualified participants were randomized to once-daily 320 mg Valsartan or 25 mg HCTZ therapy for 8 months, with both agents started at half dose and increased to full dose after the first month. Notably, one study subject did not tolerate full dose Valsartan due to relative hypotension and was; therefore, continued on half dose (Valsartan 160 mg) for the entire study duration. All study measurements and procedures were performed at baseline, 1–7 days prior to randomization and repeated at the end of the 8-month treatment period.

### Procedures

#### *Oral glucose tolerance test (OGTT)*

A standard 75 g oral glucose tolerance test was administered only at baseline to evaluate each study subject’s glycemic status according to American Diabetes Association criteria [10], and to screen for the presence of undiagnosed diabetes. The test was performed at 8:30 AM after an overnight fast (10–12 hrs) and within 10 days of all other baseline measurements. Blood was sampled at baseline: time ‘0’ and following the standard glucose drink at 15, 30, 60, 120, and 180 minutes.

#### *Frequently sampled intravenous glucose tolerance test (FSIVGTT)*

The protocol was initiated at 8:30 AM after an overnight fast (10–12 hours). Two intravenous (antecubical vein) polyethylene catheters were inserted, one for infusions of glucose and regular human insulin and another for blood sampling. A bolus of 50% dextrose solution (0.3 g/kg body weight) was injected at time 0 and a bolus of regular human insulin (0.03 U/kg body weight) was injected at 20 min. Blood samples were collected for determination of plasma glucose and insulin levels at: -15, -10, -5, -1, 2, 3, 4, 5, 6, 8, 10, 14, 19, 22, 25, 30, 40, 50, 70, 100, 140, and 180 minutes. Data were analyzed using the Millennium Minimal Model (MINMOD) [11]. We report the Acute Insulin Response to glucose (AIR_g_) – a measure of glucose stimulated insulin secretion, Insulin Sensitivity (SI), and Disposition Index (DI) – a measure of insulin secretion adjusted for the prevailing insulin sensitivity which predicts progression to type 2 diabetes [11].

#### *Proton magnetic resonance spectroscopy (MRS)*

To study the role of steatosis in the clinical setting, we and others have developed non-invasive, in vivo technique that permits the precise and reproducible quantification of intracellular triglyceride in various human organs, including skeletal muscle [12-15], liver [16,17], myocardium [16,18-21], and pancreas [22]. This method offers a technological advantage as it distinguishes the large compartments of triglyceride in adipose tissue cells from the triglyceride droplets that are stored within the cytosol of parenchymal cells. This method is now widely accepted and has become extremely useful in obesity and diabetes clinical studies as these evaluations are fast, safe, and reliable. In this study, we evaluated hepatic, pancreatic, myocardial and skeletal muscle TG content using a1.5 Tesla Gyroscan Achieva whole body clinical system (Philips Medical Systems, Cleveland, USA) equipped with software for localized spectroscopy as described before [16,17].In short, high-resolution morphological images were collected to serve as a “roadmap” for selection of a testing volume of 27 cc within the upper right hepatic lobe, 2 cc within pancreatic tail, 6 cc in myocardial septum, and 1 cc within the skeletal muscle.All spectra were collected using PRESS sequence (Point RESolved Spectroscopy) for spatial localization and the signal acquisition with the following data acquisition pmeters: T_e_ = 27 ms, T_r_ = 3 s. All data were collected without water suppression. Sixteen acquisitions were averaged for liver, pancreas, and skeletal muscle, and 32 for heart. Areas of resonances from protons in water molecules and in methylenes of fatty acid chains were evaluated with line-fit procedure using a commercial software (NUTS-ACORNNMR, Freemont, CA) [14-17].

### Cardiac imaging

Dynamic cine images were used to quantify left ventricular (LV) volume [16,18,19]. Image analysis was performed by an observer blinded to the subject’s clinical history and treatment, using a commercially available workstation (MASS, Philips Medical Systems). Endocardial and epicardial LV borders were traced manually at end diastole and end systole from short-axis slices, and the papillary muscles were excluded from the LV cavity volume. LV mass was computed as the product of end-diastolic LV volume and myocardial density (1.05 g/mL). The fraction of blood pumped out of the left ventricle with each heart beat, the ejection fraction (EF), was calculated as the difference between left ventricular end diastolic volume and left ventricular end systolic volume divided by left ventricular end diastolic volume. EF was used as an index of global LV function.

### Abdominal MRI

The amount of subcutaneous and visceral abdominal fat was determined from a single abdominal axial image at the level between vertebral bodies L2 and L3 [23]. The image analysis was performed by a single observer who was blinded to the volunteer’s treatment, using commercially available software (MASS, Philips Medical Systems) that maps the subcutaneous and intra-abdominal adipose tissue compartments.

### Laboratory measurements

All blood was processed immediately and was analyzed within 7 days. Lipid profile, liver function tests, glucose, and insulin were analyzed in a commercial laboratory, Quest Diagnostics, Irving, TX. HbA1c was analyzed by HPLC at UT Southwestern Medical Center.

### Clinical measurements

Blood pressure was measured with a Space Labs continuous home monitor for at least a 24 hr period. The average of all results obtained during this monitoring period is reported. Waist circumference was measured at the level of the umbilicus in neutral respiratory position, using the same standard tape for all measurements during the entire study. Hip circumference was measured at the widest part of the hips.

### Statistics

Responses to therapies, measured as a difference between baseline and end of the study, were compared between the groups. The tests for normality (Shapiro-Wilk, chi-square and Kolmogorov-Smirnov) showed that the hepatic triglyceride content (hTG) response to HCTZ and disposition index - DI response to Valsartan were not normally distributed with 95% of confidence based on the results of at least one of the listed tests. All other responses, including SI response to both treatments were normally distributed with 95% confidence. Therefore we used two sample t-test for comparison of the central tendency for SI between the two groups. The F-test was used to compare the variability of the responses between the two groups. We used non-pmetric Wilcoxon-Mann–Whitney test to compare hTG and DI responses. Data were analyzed with Statgraphics Centurion XVI software. Statistical significance was set at p < 0.05.

## Results

**Figure 2:**
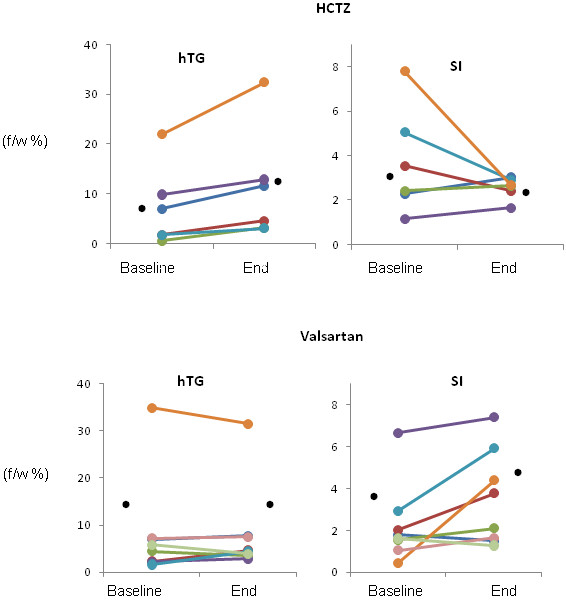
**Impact of hydrochlorothiazide (HCTZ) and Valsartan treatments on hepatic triglyceride levels (hTG) and insulin sensitivity (SI).** Results of hTG and SI are color coded relative to patient. Black points represent the averages.

**Figure 3:**
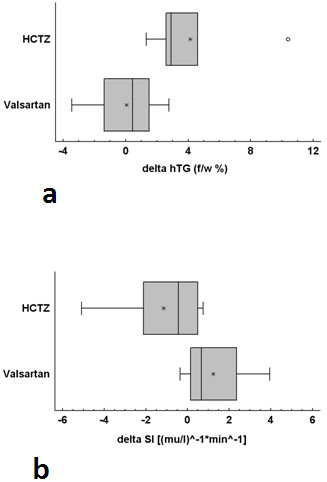
**(a) Changes in hepatic triglyceride content (hTG) after treatment with Valsartan and HCTZ ( p = 0.0098). (b)** Changes in insulin sensitivity (SI) after treatment with HCTZ and Valsartan (p = -0.0345).

The characteristics of study participants and the main study results are listed in Table 1 and Table 2 respectively. Demographic, clinical, and biochemical characteristics of both groups were similar at baseline and did not change following either treatment. However, hepatic TG levels, a measure of hepatic steatosis, increased by 57% after HCTZ (baseline average hTG = 7.18% +/- 3.30%, hTG range 0.59% - 21.97%; post HCTZ average hTG = 11.30% +/- 4.56%, hTG range 3.13% - 32.37%; and ΔhTG _HCTZ_ = 4.12%). Hepatic TG levels were unchanged after Valsartan therapy (baseline average hTG = 8.21% +/- 3.90%, hTG range 1.62% - 34.92%; post Valsartan average hTG = 8.27% +/- 3.38%, hTG range 2.83% - 31.49%, and ΔhTG _V_ = 0.06%). The increased inhepatic TG levels in the HCTZ group were accompanied by a reduction in insulin sensitivity (baseline average SI = 3.71 +/- 0.98, SI range 1.17 - 7.78; post HCTZ average SI = 2.57 +/- 0.20, SI range 1.66-3.04; and ΔSI _HCTZ_ = -1.14). Treatment with Valsartan resulted in improved insulin sensitivity (baseline average SI = 2.27 +/- 0.68, SI range 0.46- 6.67; post Valsartan average SI 3.51 +/- 0.80, SI range 1.28-7.42; and ΔSI _V_ = 1.24). Treatment-induced changes in hepatic TG levels and insulin sensitivity were statistically significant between groups (p_hTG_ = 0.0098and p_SI_ = 0.0345 respectively). The individual results as well as the changes in hTG and SI are shown in Figures 2 and 3.

**Table 1:** Characteristics of study participants (mean ± standard error)

	**HCTZ**		**Valsartan**	
**Variable**	**Baseline**	**End**	**Baseline**	**End**
**N**	6		9	
**Male**, %	**71**		**22**	
**Age**, years	**35** ± 11		**37** ± 5	
**Hip** circumference, cm	**110** ± 6	**115** ± 9	**116** ± 10	**116** ± 10
**Waist** circumference, cm	**101** ± 12	**104** ± 16	**107** ± 9	**107** ± 11
**BMI**, kg/m^2^	**30.7** ± 2.4	**31.6** ± 2.7	**34.7** ± 3.4	**34.9** ± 3.9
**SBP**, mmHg	**120** ± 11	119 ± 9	**114** ± 8	**108** ± 9
**DBP**, mmHg	**72** ± 8	71 ± 5	**71** ± 7	**67** ± 5
**HR**, beat/min	**74** ± 9	**77** ± 8	**80** ± 7	**81** ± 7
**Glucose**, mg/dL	**95** ± 11	**98** ± 11	**100** ± 10	**94** ± 11
**Insulin**, /mL	**7** ± 6	**8** ± 4	**7** ± 5	**9** ± 7
**Cholesterol**, mg/dL	**209** ± 17	**217** ± 34	**194** ± 40	**192** ± 30
**Triglycerides**, mg/dL	**182** ± 118	**186** ± 52	**153** ± 106	**151** ± 83
**HDL**, mg/dL	**40** ± 8	**41** ± 6	**47** ± 14	**49** ± 20
**LDL**, mg/dL	**142** ± 17	**139** ± 32	**119** ± 31	**113** ± 27
**HbA1c**, %	**5.4** ± 0.1	**55** ± 0.3	**5.4** ± 0.5	**55** ± 0.3
**ALT**, u/L	**31** ± 8	**31** ± 12	**27** ± 25	**22** ± 15
**AST**, u/L	**29** ± 8	**26** ± 6	**21** ± 11	**19** ± 6

**Table 2:** **Hepatic triglyceride content (hTG), insulin secretion (SI) glucose stimulated insulin response (AIR**_**g**_**) and disposition index (DI) following the eight months treatment with either hydrochlorothiazide (HCTZ) or Valsartan (mean ± standard error)**

	**HCTZ**		**Valsartan**	
**Variable**	**Baseline**	**End**	**Baseline**	**End**
**hTG,** f/w, %	**7.18** ± 3.30	**11.53** ± 4.56	**8.29** ± 3.90	**8.27** ± 3.38
**SI,** (x10^-5^/min pmol/l)	**3.72** ± 0.98	**2.57** ± 0.02	**2.27** ± 0.68	**3.51** ± 0.8
**AIR**_**g**_**,** pmol/l	**445** ± 291	**466** ± 263	**701** ± 608	**708** ± 405
**DI,** (x10^-5^/min)	**1332** ± 399	**1191** ± 312	**1016** ± 157	**2034** ± 566

Of note: *DI*, SI*AIRg and describes the progression to type 2 diabetes when lowered or improvement of metabolic status when raised [11].

DI remained unchanged after HCTZ treatment (baseline average DI = 1332 +/- 399, DI range 471–3255; post HCTZ average DI = 1191 +/- 312, DI range 372–2339; and ΔDI _HCTZ_ = -141) but DI increased by a factor of 2 after treatment with Valsartan (baseline average DI = 1016 +/- 157, DI range 473–1819; post Valsartan average DI 2034 +/- 556, DI range 737 – 5739; and ΔDI _V_ =1018) – primarily due to improvement in SI. However, there change between treatment groups was not significant. Abdominal (visceral) and subcutaneous fat mass as well as myocardial structure and function remained unchanged in both therapeutic arms. Additionally, we did not detect significant changes in myocardial, pancreatic, and skeletal muscle triglyceride deposits with these interventions.

## Discussion

We have demonstrated a differential metabolic effect of two frequently prescribed antihypertensive agents in individuals at risk for type 2 diabetes. The findings of our study relate to hepatic steatosis and insulin sensitivity. HCTZ treatment worsened hepatic steatosis measured as hepatic TG content and reduced insulin sensitivity. Valsartan treatment did not affect hepatic TG levels and improved insulin sensitivity. The results of this study reinforce the message that in patients at risk for type 2 diabetes it is particularly important to choose an antihypertensive regimen that lowers blood pressure without exacerbating patient’s metabolic profile.

Our results pllel the earlier reported findings from the MEDICA study [24]. MEDICA investigators reported similar worsening in hepatic triglyceride content and insulin sensitivity following 3 months of treatment with 50 mg of HCTZ and observed no change of hepatic triglyceride levels following treatment with another ARB class medication Candesartan. In light of such tight agreement of two independent studies, therapy with HCTZ - widely accepted as first-line agent for treating hypertension - should be re-evaluated. HCTZ offer affordable and efficient blood pressure lowering but come with the added cost of worsening metabolic profiles for diabetes. This can occur even at doses of 25 mg and is sustained over a longer treatment period (8 months). By the same means, the ARB agents improve insulin sensitivity and did not cause fatty liver.

Primum non nocere - our treatment choices should not only improve the primary condition for which they are prescribed, but we must ensure that our patients suffer no harm. The ultimate goal of any antihypertensive therapy is to prevent cardiovascular events. The use of an antihypertensive agent that worsens hepatic steatosis and insulin resistance, both of which promote cardiovascular disease, negates the ultimate cardiovascular-preventive goal of the treatment. Hypertension clusters with metabolic syndrome, diabetes, and hepatic steatosis, and requires life-long pharmacologic treatment. When all aspects are balanced i.e. the blood pressure lowering effect and the worsening metabolic profile the use of HCTZ as a first line therapeutic choice should be questioned.

The underlying mechanism of the deleterious metabolic action of HCTZ is still debated although our results implicate concomitant worsening of fatty liver and decreased insulin sensitivity. Chronic exposure to angiotensin II may render fat cells less efficient in their capacity to adequately store excess triglyceride, resulting in tissue overflow with ectopic triglyceride and ultimately hepatic steatosis. Interestingly, blocking the renin-angiotensin system by Valsartan did not result in fatty liver. The favorable metabolic action of Valsartan is probably complex and could originate from improvement of insulin sensitivity [8] or it could be facilitated through the recruitment and differentiation of adipocytes [25]. In light of the above-mentioned observations it seems natural to hypothesize that pairing ARB with HCTZ could block angiotensin II and mitigate the adverse metabolic effects induced by HCTZ. Regrettably, an attempt to block these unfavorable metabolic effects of HCTZ by combining it with losartan was not successful [26].

We acknowledge that this study is small and these results should be replicated, yet the effect size was considerable and very compble to that found in a similar independently conducted study [24]. We also note that small sample size did not allow us to further explore the mechanisms that contribute to our observations.

## Conclusions

We have documented that HCTZ therapy leads to the development of hepatic steatosis and compromised insulin sensitivity in subjects at high risk for type 2 diabetes. Conversely, treatment with Valsartan did not cause ectopic fat redistribution, and in fact, leads to improved insulin sensitivity. Further studies are needed to determine the exact mechanism by which HCTZ exerts its deleterious effects and whether these changes in metabolic pmeters will translate into an increase in major long-term adverse cardiovascular events. While the clinical relevance of thiazide-induced metabolic derangements remains uncertain at this time, hypertensive individuals at risk for diabetes and those with known hepatic steatosis should opt for antihypertensive agents that are metabolically benign – i.e. ARB or ACE inhibitors – until this issue is clarified.

## Competing interests

This study was funded as the investigator initiated research award to LSS and RGB by Novartis.

## Authors’ contributions

ALP and IL designed and carried OGTT and FSIVGTT experiments. Participated in results interpretation and writing the manuscript. EWS processed MR imaging and spectroscopy data, participated in data interpretation, and assisted with statistical analysis.JW assisted in GCRC experiments, created and maintained data base for the study, processed FSIVGTT data using MinMode software, contributed to discussion on results interpretation.RGV participated in study design and results interpretation.LSS designed the study, overlooked all study procedures, executed MR Spectroscopy and wrote the paper. All authors read and approved the final manuscript.
